# σ^S^-Mediated Stress Response Induced by Outer Membrane Perturbation Dampens Virulence in *Salmonella enterica* serovar Typhimurium

**DOI:** 10.3389/fmicb.2021.750940

**Published:** 2021-09-30

**Authors:** Seul I Kim, Eunsuk Kim, Hyunjin Yoon

**Affiliations:** ^1^Department of Molecular Science and Technology, Ajou University, Suwon, South Korea; ^2^Department of Applied Chemistry and Biological Engineering, Ajou University, Suwon, South Korea

**Keywords:** *Salmonella* Typhimurium, RpoS (σ^S^), *ssrA*, *Salmonella* pathogenicity island-2, virulence

## Abstract

*Salmonella* alters cellular processes as a strategy to improve its intracellular fitness during host infection. Alternative *σ* factors are known to rewire cellular transcriptional regulation in response to environmental stressors. σ^s^ factor encoded by the *rpoS* gene is a key regulator required for eliciting the general stress response in many proteobacteria. In this study, *Salmonella* Typhimurium deprived of an outer membrane protein YcfR was attenuated in intracellular survival and exhibited downregulation in *Salmonella* pathogenicity island-2 (SPI-2) genes. This decreased SPI-2 expression caused by the outer membrane perturbation was abolished in the absence of *rpoS*. Interestingly, regardless of the defects in the outer membrane integrity, RpoS overproduction decreased transcription from the common promoter of *ssrA* and *ssrB*, which encode a two-component regulatory system for SPI-2. RpoS was found to compete with RpoD for binding to the P*_ssrA_* region, and its binding activity with RNA polymerase (RNAP) to form Eσ^s^ holoenzyme was stimulated by the small regulatory protein Crl. This study demonstrates that *Salmonella* undergoing RpoS-associated stress responses due to impaired envelope integrity may reciprocally downregulate the expression of SPI-2 genes to reduce its virulence.

## Introduction

The bacterial RNA polymerase (RNAP) holoenzyme is a provisional complex between a multi-subunit RNAP core enzyme (E, α_2_ββ'ω) and an σ factor. The σ factor forming the Eσ complex directs promoter-specific transcription initiation and then dissociates from the core enzyme E after transcription initiation. In many proteobacteria, σ^D^ (σ^70^), encoded by *rpoD*, functions as a major σ subunit responsible for the transcription of constitutive promoters. Under unfavorable conditions, bacteria exploit alternative σ factors to redistribute RNAP core enzyme specificity toward discrete subsets of genes whose products help survive and adapt to environmental stressors (Bang et al., 2005). *Salmonella enterica* serovar Typhimurium possesses five alternative σ factors, including extra-cytoplasmic stress-specific σ^E^ (σ^24^, RpoE), flagella-chemotaxis-specific σ^F^ (σ^28^, FliA), heat-shock response-specific σ^H^ (σ^32^, RpoH), stationary-phase nutrient-starvation-specific σ^S^ (σ^38^, RpoS), and nitrogen-starvation-specific σ^N^ (σ^54^, RpoN). The abundance of alternative σ factors available for Eσ complex formation is regulated not only by environmental signals (Shimada et al., 2017) but also by the interaction with two types of inhibitory proteins, anti-σ factors, and adaptor proteins (Trevino-Quintanilla et al., 2013). Different σ factors operate discrete regulatory circuits containing cognate genes and operons in response to specific environmental cues, but some transcriptional regulons are coordinated by multiple σ factors that function in a regulatory cascade or by competitive interactions. In response to the diverse stimuli encountered by bacterial pathogens upon host infection, multiple alternative σ factors interact with each other to promote bacterial adaptation in hostile conditions. σ^E^ can activate one of the *rpoH* promoters (Hiratsu et al., 1995; Vanaporn et al., 2008) and σ^H^, in turn, stimulates the transcription of Hfq (Muffler et al., 1996), which is required for efficient translation of *rpoS* mRNA (Bang et al., 2005), indicating sequential activation of multiple regulons by a regulatory cascade of σ^E^, σ^H^, and σ^S^ under certain circumstances. Besides, there is a trade-off between self-preservation and nutritional competence and genes required for membrane integrity maintenance and genes associated with metabolism are reciprocally controlled by competitive action between σ factors (Ferenci, 2005; Levi-Meyrueis et al., 2015). In the context of competitive action between σ factors, σ^70^ and σ^S^ recognize almost identical −35 and −10 promoter elements, especially the −10 region (Hengge-Aronis, 2002b). Therefore, competitive binding of Eσ^S^ to the overlapping promoter regions may occlude transcription initiation by Eσ^70^, inducing the transcription of a repertoire of genes by σ^S^ under stressful environments (Levi-Meyrueis et al., 2015).

Many genes whose promoters bind to both σ^70^ and σ^S^ show stronger transcription activities with σ^70^ binding than with σ^S^ binding, implying a negative role of σ^S^ in gene expression (Levi-Meyrueis et al., 2015; Grove et al., 2017; Yin et al., 2018). Interestingly, nullifying the negative effects of σ^S^ is beneficial to bacterial growth in the absence of environmental stressors (Zambrano et al., 1993; Notley-McRobb et al., 2002). The attenuated expression associated with Eσ^S^ may confer fitness advantages to bacteria during unfavorable conditions. σ^S^ is induced under nutrient-depleted stationary phase or in response to various stressors, and its activity in *Salmonella* is known to alter transcription or protein production of more than 20% of its genome (Levi-Meyrueis et al., 2014; Lago et al., 2017). σ^S^ upregulates or downregulates the expression of a myriad of genes involved in carbohydrate and amino acid metabolism, stress resistance, and membrane integrity directly or indirectly. In contrast to the essential roles of σ^S^ in bacterial stress resistance, the requirement of σ^S^ for bacterial virulence varies between bacterial species (Dong and Schellhorn, 2010). *Salmonella* Typhimurium lacking *rpoS* gene showed reduced virulence, and σ^S^ factor was found to activate the transcription of *spvR* and *spvABCD* virulence plasmid genes (Fang et al., 1992; Kowarz et al., 1994).

In this study, we induced outer membrane perturbation on *S. Typhimurium* by deleting *ycfR* to stimulate σ^S^-mediated adaptation responses. YcfR is a putative outer membrane protein that is expressed under stressful conditions in enteric pathogens and is known as a multiple stress resistance protein (Zhang et al., 2007; Salazar et al., 2013). Our previous study demonstrated that the deletion of *ycfR* caused structural alterations in lipopolysaccharide and destabilized *Salmonella* envelope integrity (Kim and Yoon, 2019). *Salmonella* devoid of YcfR tremendously increased *rpoS* transcription and showed an increase in curli fibers, cellulose, and c-di-GMP production and a decrease in motility, implicating comprehensive transcriptional alterations by σ^S^ in response to stress on the cellular envelope (Kim and Yoon, 2019). Besides the known repertoires of σ^S^ regulatory circuits, such as biofilm formation, this study revealed that virulence genes of *Salmonella* pathogenicity island-2 (SPI-2) were downregulated by σ^S^. SPI-2 is a locus responsible for the type III secretion system (T3SS) injectisome-mediated delivery of virulence factors from *Salmonella* to host cells and is critical for bacterial survival and replication inside host cells (Jennings et al., 2017). The negative role of σ^S^ in SPI-2 regulation was influenced by a small regulatory protein Crl. Crl is known to be required for σ^S^-dependent transcriptional initiation at the promoters of *adrA* and *csgD* genes, whose products activate curli and cellulose production (Robbe-Saule et al., 2006). The role of σ^S^ in *Salmonella* virulence regulation was elucidated by examining the interaction between σ^S^ and the *ssrAB* promoter, which encodes the two-component regulatory system SsrAB for SPI-2.

## Materials and Methods

### Bacterial Strains, Plasmids, and Growth Conditions

*Salmonella enterica* serovar Typhimurium ATCC 14028 was used as the parent strain. *Salmonella* mutants of Δ*ycfR* and Δ*rpoS* were constructed using the phage lambda (λ) Red recombination system as described in previous studies (Yoon et al., 2009; Kim and Yoon, 2019) and a mutant lacking both *ycfR* and *rpoS* was constructed using P22 HT105/1 int-201-mediated transduction (Kwoh and Kemper, 1978). The phage λ Red recombination system was also used for the construction of *Salmonella* strains producing HA-tagged SPI-2 proteins (SseC and SsaN) as described in the previous study (Kim et al., 2018). In brief, the kanamycin resistance (*kan*) cassette of pKD13-2HA was amplified by PCR using primers designed to provide 40-nucleotide sequences homologous to target genes at both termini of the resultant PCR products. The PCR products were introduced into *Salmonella* cells harboring pKD46 to insert the HA-coding sequences with a *kan* cassette prior to the stop codon sequences. The *kan* marker was subsequently removed using pCP20 providing a flip recombinase. Primers used for the construction of HA-tagged SPI-2 genes are listed in Supplementary Table 1. *Escherichia coli* DH5α strain was used for plasmid cloning and protein purification.

To express *rpoS* in *trans*, the *rpoS* gene was cloned into pACYC184 (Chang and Cohen, 1978) and pBbA2sk-RFP vectors (Lee et al., 2011). For the construction of pRpoS, the *rpoS* CDS and its promoter region were amplified by PCR using primers pRpoS-*CF* and pRpoS-CR and inserted into pACYC184 through BamHI and SalI restriction enzyme sites. In cloning pRpoS2, the *rpoS* gene was amplified using PCR with pRpoS-CF2 and pRpoS-CR2 primers, digested with EcoRI and BglII, and ligated with EcoRI/BglII digested pBbA2sk-RFP plasmid. Primer sequences are listed in Supplementary Table 1.

To construct transcriptional *lacZ* fusion to the P*_ssrA_* and P*_ssrB_* regions, the promoter regions of *ssrA* (from −253 to +209) and *ssrB* (from −90 to +303) were amplified by PCR using primers (Supplementary Table 1) of pssrA-lacZ-*CF* and pssrA-lacZ-CR for *ssrA* and primers (Supplementary Table 1) pssrB-lacZ-*CF* and pssrB-lacZ-CR for *ssrB*, as described by Feng et al. (2003). The amplified promoter regions were cloned into the pRS415 plasmid (Simons et al., 1987) using EcoRI and SalI restriction enzyme sites.

RpoS, RpoD, and Crl proteins were tagged with His_6_ at their N-termini by cloning three genes into the pUHE21-lacI^q^ plasmid (Soncini et al., 1995) *via* EcoRI and HindIII and inducing their expression using IPTG. The primers used for the construction of His_6_ tagged proteins are listed in Supplementary Table 1. All restriction enzymes and ligases were purchased from Takara Bio, Inc. (CA, United States).

*Salmonella* cells were cultivated in Luria-Bertani (LB) medium broth or acidic minimal medium (AMM) broth at 220rpm at 37°C, as described in previous studies (Yoon et al., 2009, 2011). For AMM cultivation, bacterial cells at the stationary growth phase in LB medium broth were washed twice with PBS, diluted in pH 7.0 minimal medium broth at a 1:100 ratio, and cultivated overnight. Pre-cultured *Salmonella* cells in minimal medium broth (pH 7.0) were diluted in minimal medium broth (pH 5.0) at a 1:20 ratio and cultivated for 3h to mimic intracellular conditions (Yoon et al., 2009). Antibiotics were purchased from Sigma-Aldrich (MO, United States) and used when required: ampicillin (Amp, 50μg/ml), chloramphenicol (Cm, 35μg/ml), kanamycin (Kan, 50μg/ml), and anhydrotetracycline (aTc, 0.2 or 0.5ng/ml).

### Mammalian Cell Infection

To assess bacterial invasiveness, HeLa human epithelial cell line (ATCC CCL-2) was infected as described in the previous study (Kim et al., 2018). HeLa cells were seeded in 24-well plate at 2×10^5^ cells/well and incubated in Dulbecco’s modified Eagle’s medium (DMEM; Corning cellgro, Thermo Scientific Inc., IL, United States) supplemented with 4.5g/L glucose (Thermo Scientific Inc.) and 10% fetal bovine serum (FBS; Gibco, Thermo Scientific Inc.) at 37°C with 5% CO_2_. After overnight incubation, HeLa cells were treated with *Salmonella* cells grown for 2.5h in LB medium broth at a multiplicity of infection (MOI) of 100 and centrifuged at 500×*g* for 5min. At 30min post-infection, the infected cells were washed twice with PBS and replenished with fresh DMEM containing 100μg/ml gentamicin for 1.5h to remove extracellular *Salmonella* cells. The infected HeLa cells were washed three times with PBS and lysed with 1% Triton X-100. The cell lysates were diluted and plated on LB agar to count intracellular *Salmonella* cells.

*Salmonella* survival inside macrophages was examined as described elsewhere (Yoon et al., 2009; Kim and Yoon, 2019). Murine macrophage RAW264.7 (ATCC TIB-71) cells were seeded in 24-well plate at 5×10^5^ cells/well and incubated in DMEM containing 4.5g/L glucose and 10% FBS at 37°C with 5% CO_2_ overnight. Monolayered-macrophage cells were infected with *Salmonella* cells grown overnight in LB medium broth at MOI 100, as described for HeLa cell infection. After 30min of infection, the extracellular bacteria were removed by replacing the medium with DMEM containing 100μg/ml gentamicin for 1.5h. The infected macrophages were washed with PBS three times and incubated in fresh DMEM containing 20μg/ml gentamicin for additional 8h. To enumerate intracellular bacteria, RAW264.7 cells were lysed, and the lysates were spread on LB agar as described above.

### qRT-PCR Analysis

Bacterial total RNA was isolated from *Salmonella* cultivated in LB medium and AMM broth or RAW264.7 cells infected with *Salmonella*. Bacterial cells cultivated *in vitro* were treated with RNAprotect Bacteria Reagent (Qiagen, Hilden, Germany) and subjected to total RNA extraction using RNeasy mini kit (Qiagen). For RNA extraction from intracellular bacteria, infected macrophage cells were treated with RNAlater™ Stabilization Solution (Invitrogen, Thermo Scientific Inc.) and processed with RNeasy mini kit according to the manufacturer’s recommendations. Isolated total RNA was treated with RNase-free DNase (Ambion, TX, United States) at 37°C for 30min and used to synthesize cDNA using RNA to cDNA EcoDryTM Premix (Takara Bio United States, Inc.). cDNA corresponding to 10ng of input RNA was used as a template in each qRT-PCR, and the primer sequences are listed in Supplementary Table 2. qRT-PCR was conducted using the StepOnePlus Real-time PCR system (Applied Biosystems, MA, United States) with Power SYBR Green PCR Master Mix (Applied Biosystems), and the levels of amplified PCR products were normalized to those of *gyrB* (Yoon et al., 2009).

### β-Galactosidase Assay

The β-galactosidase assay was conducted using the Miller method (Smale, 2010). Bacterial cells were cultivated in LB medium broth, and β-galactosidase activity normalized to the number of input bacteria was represented by Miller units. Miller units were computed as follows: Miller unit=[1,000×(OD_420_–1.75×OD_550_)]/(*t*×*V*×OD_600_), where *t* is time (min) and *V* is volume (ml).

### Immunoblot Assay

Bacterial cells were pelleted and resuspended in 1× Laemmli sample buffer (Bio-Rad Laboratories, Inc., CA, United States). The aliquots were loaded on 10% SDS-PAGE gels, and the separated proteins were transferred to PVDF membranes (Bio-Rad Laboratories, Inc.). The membrane was blocked with 5% skim milk solution and treated with anti-RpoS antibody (anti-*E. coli* RNA Sigma S antibody, BioLegend, CA, United States) at a 1:2,000 dilution ratio or anti-DnaK antibody (Enzo Life Science, NY, United States) at a 1:10,000 dilution ratio in combination with horseradish peroxidase (HRP)-conjugated secondary antibody (Bio-Rad Laboratories, Inc.) at a 1:3,000 dilution ratio. SPI-2 proteins tagged with HA were identified using anti-HA antibody (1:10,000 dilution; Sigma, United States) as a primary antibody. Immunoblotting was conducted using ECL™ Western Blotting Detection Reagents kit (GE Healthcare, Thermo Scientific Inc.), and the blot images were visualized using the ChemiDoc™ MP System (Bio-Rad Laboratories, Inc.). The intensity of the blot images was analyzed using ImageJ software.^1^

### Chromatin Immunoprecipitation Assay

The Chromatin immunoprecipitation (ChIP) assay was performed as previously described (Gu et al., 2016; Yin et al., 2018) with minor modifications. Briefly, *Salmonella* cells cultivated in the stationary growth phase in LB medium broth were fixed with 1% formaldehyde solution for 10min and subsequently treated with 100mM glycine for 5min. Cells were washed with cold PBS and resuspended in SDS lysis buffer (50mM Tris–HCl, 10mM EDTA, 1% SDS, and pH 8.0) containing 1× protease inhibitor. After 10min of incubation, the cell extract was sonicated to fragment genomic DNA into 200bp to 1kb and centrifuged at 12,000×*g* for 10min. The supernatant solution was used as input DNA, and the aliquots were further processed for pre-clearing and immunoprecipitation (IP) samples. The lysate solution containing DNA-protein complexes was pre-incubated with Protein A/G Plus-Agarose (Santa Cruz Biotechnology, Inc. TX, United States) at 4°C for 2h to remove DNA or proteins non-specifically bound to Protein A/G Plus-Agarose and centrifuged at 800×*g* for 3min. The resultant pellet fraction was used as a pre-clearing sample, and the supernatant solution was further incubated with the anti-RpoS antibody at 4°C overnight, followed by Protein A/G Plus-Agarose at 4°C for 2h, and centrifuged at 800×*g* for 3min to immunoprecipitate DNA-RpoS complexes bound to the agarose. The pellet fraction was used as an IP sample. The pre-clearing and IP samples were washed with LiCl wash buffer (100mM Tris–HCl, pH 8.0, 2% Triton X-100, and 250mM LiCl), twice with high-salt buffer (100mM Tris–HCl, pH 8.0, 600mM NaCl, and 2% Triton X-100), twice with low-salt buffer (100mM Tris–HCl, pH 8.0, 300mM NaCl, and 2% Triton X-100), and with TE wash buffer (10mM Tris–HCl, pH 8.0, and 1mM EDTA). The precipitated DNA-protein complexes were eluted with ChIP elution buffer (50mM Tris–HCl, pH 8.0, 10mM EDTA, and 1% SDS) and incubated with 0.2M NaCl at 65°C overnight to resolve DNA-protein cross-links. All samples were treated with RNase A (10mg/ml) at 37°C for 30min and further incubated with a protease solution (1M Tris–HCl, pH 8.0, 500mM EDTA, proteinase K, and 5M NaCl) at 65°C for 4h. DNA from pre-clearing and IP samples was extracted using phenol: chloroform: isoamyl alcohol (25: 24: 1) solution, precipitated with EtOH and NaOAc (pH 5.2), and resuspended in distilled water.

### ChIP-Quantitative PCR Assay

DNA cross-linked to RpoS was analyzed using quantitative PCR (qPCR), as previously described (Hermans et al., 2016). Relative enrichment (RE) of the promoter of interest was computed using differences in Ct values (ΔCt) with *gyrB* gene as an endogenous control as follows: RE=2^−(∆Ct^_IP_^−∆Ct^_Pre-clearing_^)^, where ∆Ct_IP_ is Ct_promoter test_ −Ct*_gyrB_* for the IP samples and ∆Ct_Pre-clearing_ is Ct_promoter test_ −Ct*_gyrB_* for the pre-clearing samples. Aliquots of DNA purified from IP and pre-clearing samples and serial dilutions of input DNA were used as templates in qPCR, and the qPCR primers are listed in Supplementary Table 3. Amplified PCR products were analyzed using the StepOnePlus Real-time PCR system with Power SYBR Green reagent.

### Purification of His_6_-Tagged Protein

*Escherichia coli* strains producing His_6_-tagged RpoS, RpoD, and Crl proteins were cultivated in LB medium broth, and the proteins were induced by adding 0.05mM (RpoS and RpoD) or 1mM (Crl) isopropyl β-D-1-thiogalactopyranoside for 7 or 3h at 30°C. Bacterial cells were centrifuged at 10,000×*g* for 10min and resuspended in lysis buffer (50mM NaH_2_PO_4_, 300mM NaCl, 20mM imidazole, and pH 8.0) containing 1mg/ml lysozyme. After 30min incubation on ice, the cells were sonicated and centrifuged at 10,000×*g* and 4°C for 20min. The resultant soluble lysate fraction was treated with Ni^2+^-nitrilotriacetic acid (Ni^2+^-NTA) agarose beads (Qiagen) at 4°C for 1h with rotation and loaded onto a Ni^2+^-NTA agarose affinity column (Qiagen). The column was washed with washing buffer (50mM NaH_2_PO_4_, 300mM NaCl, 40mM imidazole, and pH 8.0) three times, and the proteins were eluted with elution buffer (50mM NaH_2_PO_4_, 300mM NaCl, 300mM imidazole, and pH 8.0). The eluted protein fraction was packed into SnakeSkin™ Dialysis tubing with 10K MWCO (Thermo Scientific Inc.) and subjected to dialysis at 4°C in dialysis buffer (20mM Tris–HCl, pH 8.0, 150mM NaCl, 0.1mM EDTA, 5mM DTT, and 20% glycerol). Purified proteins were quantified using Bradford assay.

### Electrophoretic Mobility Shift Assay

The binding between σ factors and the P*_ssrA_* region was investigated using His_6_-RpoS or His_6_-RpoD in combination with His_6_-Crl. The P*_ssrA_* region was PCR-amplified using primers ssrA-electrophoretic mobility shift assay (EMSA)-F and ssrA-EMSA-R. The *csgBA* promoter region amplified using primers cgsBA-EMSA-F and cgsBA-EMSA-R was employed as a positive control, whereas the STM14_1978 (putative ABC transporter permease component) CDS region amplified using primers STM14_1978 EMSA-F and STM14_1978 EMSA-R was used as a negative control. The primer sequences used in the EMSA are listed in Supplementary Table 3. EMSA was performed as described previously (Bougdour et al., 2004; Storvik and Foster, 2010) with the following modifications. To reconstitute the RNAP holoenzyme, 20nM RNAP core enzyme (*E. coli* RNAP Core Enzyme; NEB, MA, United States) was incubated with 300nM His_6_-RpoS or His_6_-RpoD in a binding buffer (200mM Tris–HCl, pH 8.0, 30mM KCl, 10mM MgCl_2_, 50mM NaCl, 1mM DTT, 1mM EDTA, and BSA 20μg/ml) at 30°C for 45min. DNA of 20ng was incubated with the reconstituted RNAP holoenzyme in a binding buffer (50mM Tris–HCl, pH 8.0, 200mM KCl, 3mM MgCl_2_, 1mM DTT, 0.1mM EDTA, BSA μg/ml, and Poly (di-dc) 12ng/μl) at 25°C for 30min. The reactant was analyzed by electrophoresis using 5% native polyacrylamide gel, and DNA fragments were stained with EtBr solution and detected using the ChemiDoc MP System.

In the competitive binding assay between His_6_-RpoS and His_6_-RpoD, one σ factor was used at a constant concentration of 75nM and the other competitor σ factor was used at incremental concentrations from 12.5 to 150nM. After RNAP holoenzyme reconstitution with different concentrations of σ factors, DNA corresponding to the P*_ssrA_* region was added to the binding reaction and analyzed as described above. To localize His_6_-RpoS after electrophoresis on a native polyacrylamide gel, proteins on the gel were transferred to a PVDF membrane and processed as described in the immunoblot assay above. Anti-*E. coli* RNA sigma S antibody was used as a primary antibody at a 1:2,000 dilution ratio, and HRP-conjugated goat anti-mouse IgG was used as a secondary antibody at a 1:3,000 dilution ratio.

In the competition assay using His_6_-RpoS in combination with His_6_-Crl, His_6_-RpoS (25, 50, and 280nM) was pre-incubated with 280nM His_6_-Crl at 25°C for 15min and then used to compete with 50nM His_6_-RpoD in the RNAP holoenzyme reconstitution reaction. After the addition of P*_ssrA_* region, the locations of P*_ssrA_* DNA and His_6_-RpoS were identified using EtBr staining and immunoblotting methods, respectively, as described above.

### Statistical Analysis

All assays were repeated at least three times, and the average values were presented with their SDs. To determine the significant differences, Student’s *t*-test was applied, and the value of *p* was calculated.

## Results

### Outer Membrane Perturbation in Δ*ycfR* Decreased SPI-2 Expression

**Figure 1:**
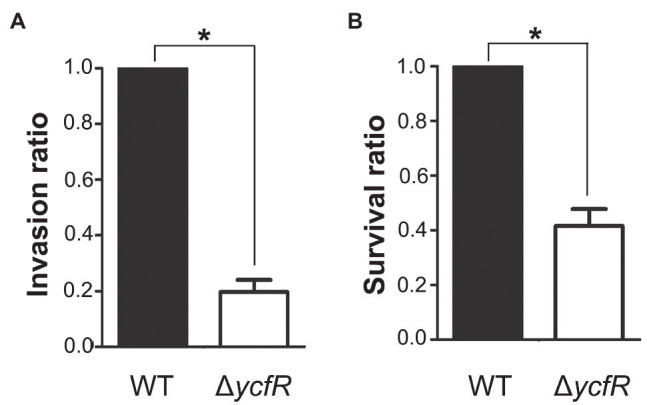
Invasion and survival of a Δ*ycfR* mutant in the host cell. **(A)** Invasive ability of wild-type and Δ*ycfR* mutant strains was assessed by infecting HeLa cells and counting intracellular bacteria 2h post-infection. The bar indicates relative invasion ability compared to wild-type *Salmonella*. **(B)** Survival inside RAW264.7 macrophages was compared between wild-type and Δ*ycfR* mutant strains 10h post-infection. The numbers phagocytosed by macrophages were found comparable between two strains (data not shown) and relative survival ratios are depicted. A significant difference (value of *p*<0.05) is denoted with an asterisk.

**Figure 2:**
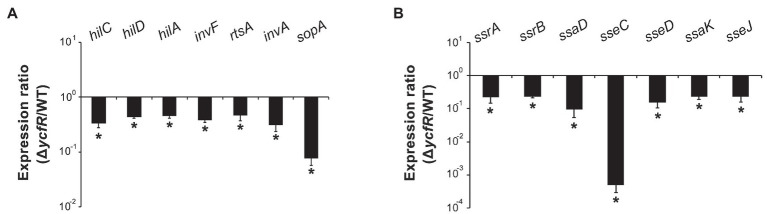
Expression of SPI-1 and *Salmonella* pathogenicity island-2 (SPI-2) genes in Δ*ycfR* mutant. **(A)** Transcription levels of SPI-1 genes were examined using RNA isolated from *Salmonella* strains grown in LB medium broth for 2h. The Ct values of qRT-PCR were normalized using those of *gyrB* and the fold-change between wild-type and Δ*ycfR* mutant strains was plotted. **(B)** To analyze the expression of SPI-2 genes, *Salmonella* strains were cultivated in LB medium broth for 10h and subjected to RNA extraction. Ct values of each gene were subtracted from those of *gyrB* for normalization, and the fold-change (Δ*ycfR*/wild-type) was calculated. An asterisk indicates a difference of a value of *p*<0.05.

YcfR, which is expressed in response to multiple stress conditions, is a putative outer membrane protein important for stress resistance in enteric pathogens such as *Salmonella* spp. and *E. coli* (Zhang et al., 2007; Salazar et al., 2013). In this study, we observed that *Salmonella* lacking YcfR was significantly attenuated in virulence during host cell infection. The lack of YcfR did not influence bacterial growth *in vitro*, but significantly reduced the ability of bacteria to invade host epithelial cells and survive inside phagocytic cells (Figure 1). The transcription of SPI-1 genes, which produce a distinct T3SS (T3SS1) and promote *Salmonella* invasion into host cells (Raffatellu et al., 2005), decreased remarkably in the Δ*ycfR* strain (Figure 2A). Besides the attenuated SPI-1 expression, the physiological changes caused by the lack of YcfR, including cellular aggregation and reduced motility (Kim and Yoon, 2019), might impair bacterial invasion ability. Interestingly, the lack of YcfR also decreased the transcription of SPI-2 genes not only inside macrophage cells (Supplementary Figure 1A) but also in LB and AMM cells *in vitro* (Figure 2B; Supplementary Figure 1B, respectively), which partially reproduce the intestinal lumen and intracellular milieux, respectively (Beuzon et al., 1999; Yoon et al., 2011).

### Downregulation of SPI-2 in Δ*ycfR* Was Attributable to RpoS

**Figure 3:**
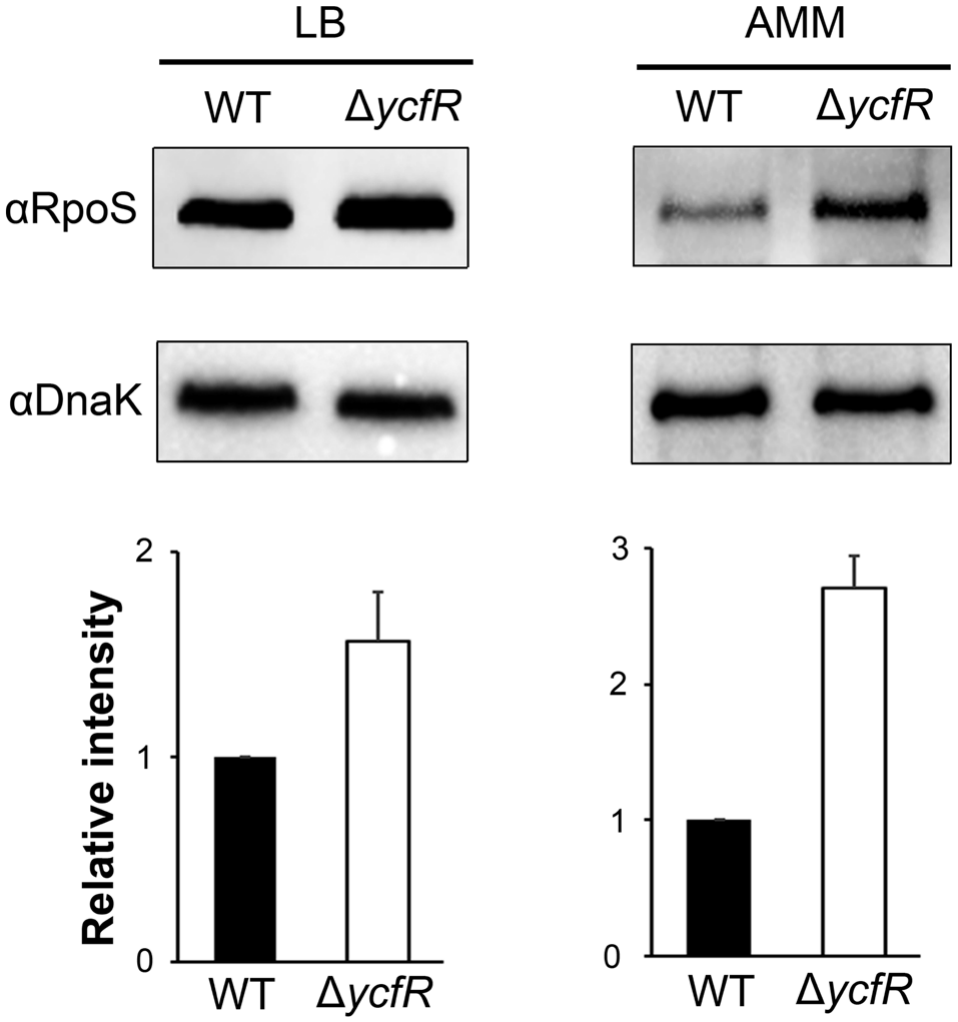
Expression of RpoS in Δ*ycfR* mutant. *Salmonella* wild-type and Δ*ycfR* mutant strains were cultivated in LB medium broth for 10h or acidic minimal medium (AMM) broth for 3h, and the expression of RpoS was compared using immunoblot assay with anti-RpoS antibody. The cytosolic protein DnaK was used as a control to standardize the protein amounts between the lanes. The abundance of RpoS was normalized to that of DnaK using ImageJ, and the ratios from three independent assays are depicted below the representative blot images.

**Figure 4:**
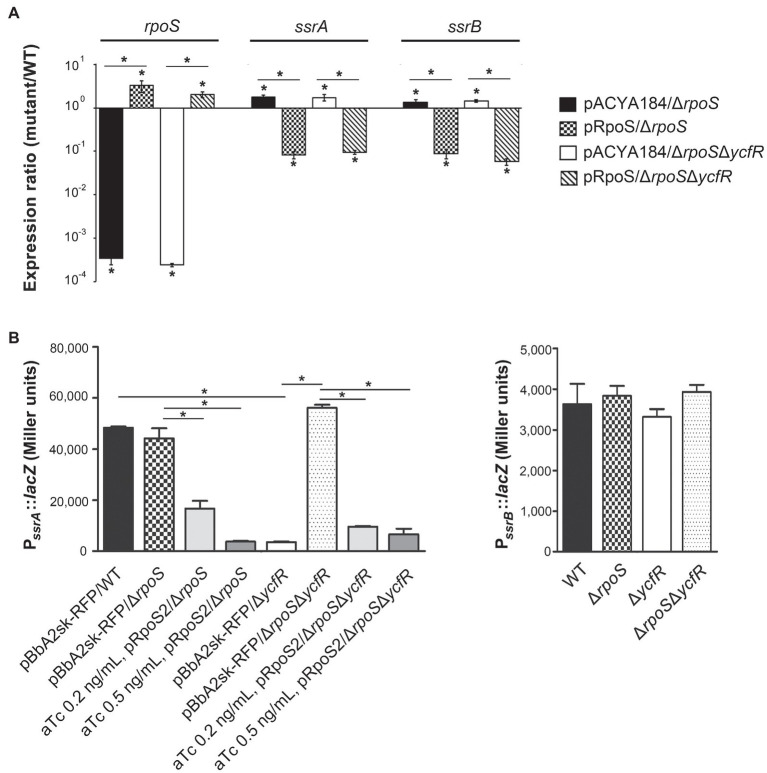
Negative regulation of *ssrA* by RpoS. **(A)** Transcription levels of *rpoS*, *ssrA*, and *ssrB* were measured using qRT-PCR. *Salmonella* strains, including wild-type, Δ*rpoS*, and Δ*rpoS*Δ*ycfR* strains, were transformed with pRpoS or pACYC184 and cultivated to the stationary growth phase in LB medium broth. Ct values of *rpoS*, *ssrA*, and *ssrB* were normalized using those of *gyrB* gene. Expression levels of *rpoS*, *ssrA*, and *ssrB* from each strain were compared with those from wild-type strain harboring pACYC184 and the fold-change (mutant/wild-type) was plotted. Value of *p* with *p*<0.05 is denoted with an asterisk. **(B)** Transcription from P*_ssrA_* and P*_ssrB_* was measured using *lacZ* transcriptional fusions. Plasmids pSsrA::*lacZ* (left) and pSsrB::*lacZ* (right) were introduced into wild-type, Δ*rpoS*, Δ*ycfR*, and Δ*rpoS*Δ*ycfR* strains. To overexpress RpoS, pRpoS2 and its empty plasmid pBbA2sk-RFP were introduced into wild-type and mutant strains, and aTc of 0.2 or 0.5ng/ml was added in the LB medium broth cultures. β-galactosidase assay was conducted with bacterial cells at the stationary growth phase. An asterisk indicates a value of *p*<0.05.

In order to figure out a transcriptional regulator that coordinates bacterial virulence in response to outer membrane perturbation, we assessed the expression of 21 regulators associated with SPI-1 or SPI-2 regulation in the Δ*ycfR* strain (Supplementary Figure 2) and found that *rpoS* showed a dramatic increase in its transcription. The levels of RpoS were compared between wild-type and Δ*ycfR* strains in LB and AMM conditions. RpoS increased in the Δ*ycfR* strain grown in both media (1.5-fold in LB; 2.7-fold in AMM; Figure 3). To examine whether an increase in RpoS could downregulate virulence genes associated with SPI-2 T3SS (T3SS2), the transcription of *ssrAB* encoding the two-component regulatory system for T3SS2 and its cognate effectors was compared. *Salmonella* deprived of RpoS slightly increased the expression of *ssrAB*, but the introduction of pRpoS expressing *rpoS* under its own promoter significantly decreased the transcription of *ssrAB*, implicating overall downregulation of their cognate T3SS2-associated genes by RpoS (Figure 4A). In addition, the decreased transcription of *ssrAB* in the absence of YcfR was nullified by the additional *rpoS* deletion, suggesting the possibility of σ^S^-mediated SPI-2 downregulation in the Δ*ycfR* strain (Figure 4A). The *ssrA* and *ssrB* genes, located adjacent to each other, encode a sensor kinase and its response regulator, respectively, and are regarded to be transcribed in a polycistronic mRNA under the same promoter (Bustamante et al., 2008; Fass and Groisman, 2009). However, the identification of a distinct promoter upstream of *ssrB* revealed the possibility that the expression of *ssrA* and *ssrB* could be uncoupled depending on the growth conditions (Feng et al., 2003, 2004). Therefore, the negative role of RpoS was reexamined using *lacZ* transcriptional fusion constructs, where the promoters of *ssrA* and *ssrB* were separately analyzed (Figure 4B). The promoter strength of *ssrA* was much stronger than that of *ssrB* in wild-type *Salmonella* harboring intact *rpoS* and *ycfR* genes, and deletion of *rpoS* alone did not alter *ssrA* or *ssrB* transcription. However, *ycfR* deletion, which led to an increase in RpoS, abolished *ssrA* transcription but not *ssrB*, and the additional *rpoS* deletion in Δ*ycfR* mutant derepressed *ssrA* only, indicating differential regulation of *ssrA* and *ssrB* by σ^S^ (Figure 4B). Again, overexpression of RpoS by the introduction of pRpoS2 decreased P*_ssrA_*::*lacZ* expression in proportion to aTc concentration. These results suggest that σ^S^ at high concentrations dampen transcription activity at the promoter region upstream of *ssrAB*.

### σ^S^ Binds Directly to the *ssrA* Promoter Region

**Figure 5:**
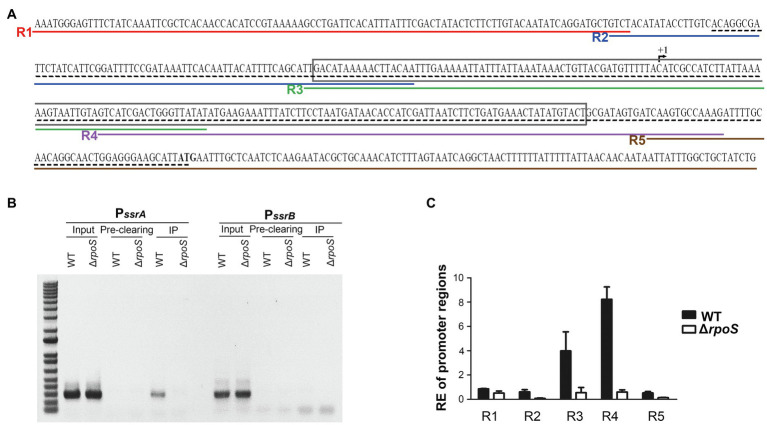
Binding of σ^S^ to the *ssrA* promoter. **(A)** Scheme for Chromatin immunoprecipitation (ChIP)-PCR and ChIP-quantitative PCR (qPCR). The +1 site of *ssrA* transcription is indicated with a broken arrow and its start codon is in bold. The region amplified by ChIP-PCR is underlined with a black dashed line and that amplified and used in electrophoretic mobility shift assay (EMSA) is boxed. Five regions amplified in ChIP-qPCR are underlined in different colors: R1 in red by primers R1-F and R1-R, R2 in blue by primers R2-F and R2-R, R3 in green by primers R3-F and R3-R, R4 in purple by primers R4-F and R4-R, and R5 in brown by primers R5-F and R5-R. **(B)** DNA-protein cross-links were fixed in *Salmonella* wild-type and Δ*rpoS* mutant strains using formaldehyde. DNA fragments bound to σ^S^ were immunoprecipitated using anti-RpoS antibody and subjected to PCR using primers specific to the P*_ssrA_* (PssrA-ChIP-F/R) and P*_ssrB_* (PssrB-ChIP-F/R) regions. Input total DNA and pre-clearing (DNA non-specifically bound to Protein A/G agarose) were analyzed in parallel. The ChIP-PCR products were separated by 1% agarose gel electrophoresis. **(C)** DNA fragments precipitated in IP and pre-clearing were used as templates in ChIP-qPCR using five different primer sets (R1-F/R, R2-F/R, R3-F/R, R4-F/R, and R5-F/R). Enrichment of the P*_ssrA_* region with σ^S^ was relatively computed using the following formula: relative enrichment (RE)=2^−(∆Ct^_IP_^−∆Ct^_Pre-clearing_^)^, where ∆Ct_IP_ is Ct_P*ssrA*_ −Ct *_gyrB_* for the IP samples and ∆Ct_Pre-clearing_ is Ct_P*ssrA*_ −Ct*_gyrB_* for the pre-clearing samples. *gyrB* was used as an endogenous control.

To examine whether σ^S^ directly controls the transcription of *ssrA*, a ChIP assay was performed on *Salmonella* cells in the stationary growth phase using σ^S^ as a bait. DNA fragments bound to σ^S^ were co-precipitated using anti-RpoS antibody and used as templates in PCR using primers targeting the promoter regions of *ssrA* and *ssrB* (Figure 5A; Supplementary Figure 2). DNA fragments containing the P*_ssrA_* region were bound to σ^S^ and amplified by PCR, but the P*_ssrB_* region did not co-precipitate with σ^S^ (Figure 5B). When five different primer sets from R1-F/R to R5-F/R were used to dissect the *ssrA* promoter region, only two primer sets, R3-F/R and R4-F/R, resulted in significant PCR amplification (Figure 5C), inferring that σ^S^ binds to DNA sequences covering −61 to +136bp at least from the transcription start site of *ssrA* (Feng et al., 2003). It is believed that the *ssrA* promoter requires RNAP holoenzyme harnessing σ^70^, and the consensus −10 and −35 regions for σ^70^ binding were also predicted (Ramachandran et al., 2012; Banda et al., 2019). Our results raised the possibility that the *ssrA* promoter could recruit σ^S^ as well as σ^70^. The possibility of σ^S^ binding to the P*_ssrA_* region was also proposed *in silico* in a previous study (Ramachandran et al., 2012). We further investigated transcription initiation at P*_ssrA_*, which is controlled by mechanical interaction with σ factors.

### σ^S^ Competes With σ^70^ for Binding to the *ssrA* Promoter Region

**Figure 6:**
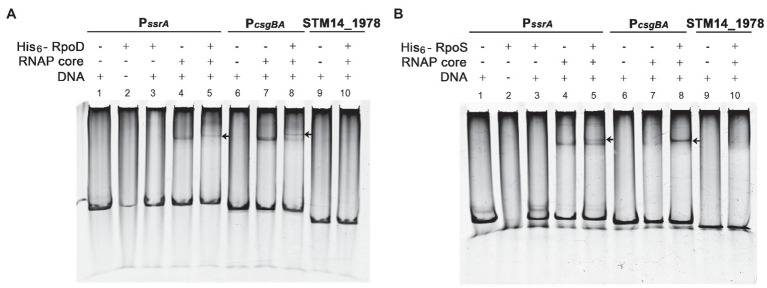
Interaction of P*_ssrA_* with either σ^S^ or σ^70^ *in vitro*. Binding of His_6_-RpoD **(A)** and His_6_-RpoS **(B)** to the *ssrA* promoter region was tested *in vitro* using EMSA. DNA fragments of the P*_ssrA_* region (178bp), P*_csgBA_* region (158bp; a positive control), and STM14_1978 CDS region (148bp; a negative control) were incubated with RNA polymerase (RNAP) core enzyme in combination with His_6_-RpoD or His_6_-RpoS. The DNA-protein complexes were loaded onto a 5% native polyacrylamide gel and stained with EtBr. An arrow indicates a shifted band comprising P*_ssrA_* or P*_csgBA_* DNA cross-linked with the RNAP holoenzyme.

The promoter recognition sequences for σ^S^ and σ^70^ are nearly identical, and a strong functional similarity between σ^S^ and σ^70^ has been suggested. Many σ^S^-regulated genes, such as the *csgBA* operon, can be transcribed by either σ^S^ or σ^70^ *in vitro* (Arnqvist et al., 1994; Typas et al., 2007b). The possibility of biphasic *ssrA* transcription initiation by σ^S^ and σ^70^ was examined *in vitro*. A DNA fragment of 172bp encompassing the P*_ssrA_* region targeted by *ssrA* regulators was incubated with each σ factor (His_6_-RpoS or His_6_-RpoD) in the presence or absence of RNAP core enzyme E. The P*_csgBA_* region recognized by either σ^S^ or σ^70^ was used as a positive control, while a DNA fragment of the STM14_1978 gene devoid of the canonical sequences recognized by σ^S^ and σ^70^ was used as a negative control. The core enzyme alone could form a complex with DNA fragments of the P*_ssrA_* or P*_csgBA_* regions in a non-specific manner, as predicted elsewhere, and the addition of either σ factor (σ^S^ or σ^70^) retarded the mobility of the DNA-protein complex. This indicated that σ factor was engaged in the complex formation between RNAP holoenzyme (Eσ; α_2_ββ′σω) and the DNA fragments (Figure 6). Interestingly, the addition of σ^S^ produced two bands, presumably a lower one between the core enzyme E and the P*_ssrA_* and an upper one between the Eσ^S^ and the P*_ssrA_*, whereas σ^70^ incorporation produced a single shifted band, representing robust complex formation between Eσ^70^ and P*_ssrA_* (compare Figure 6A, lane 5 and Figure 6B, lane 5). These results stimulated us to compare the binding affinities between Eσ^S^ and Eσ^70^ at the P*_ssrA_* region.

**Figure 7:**
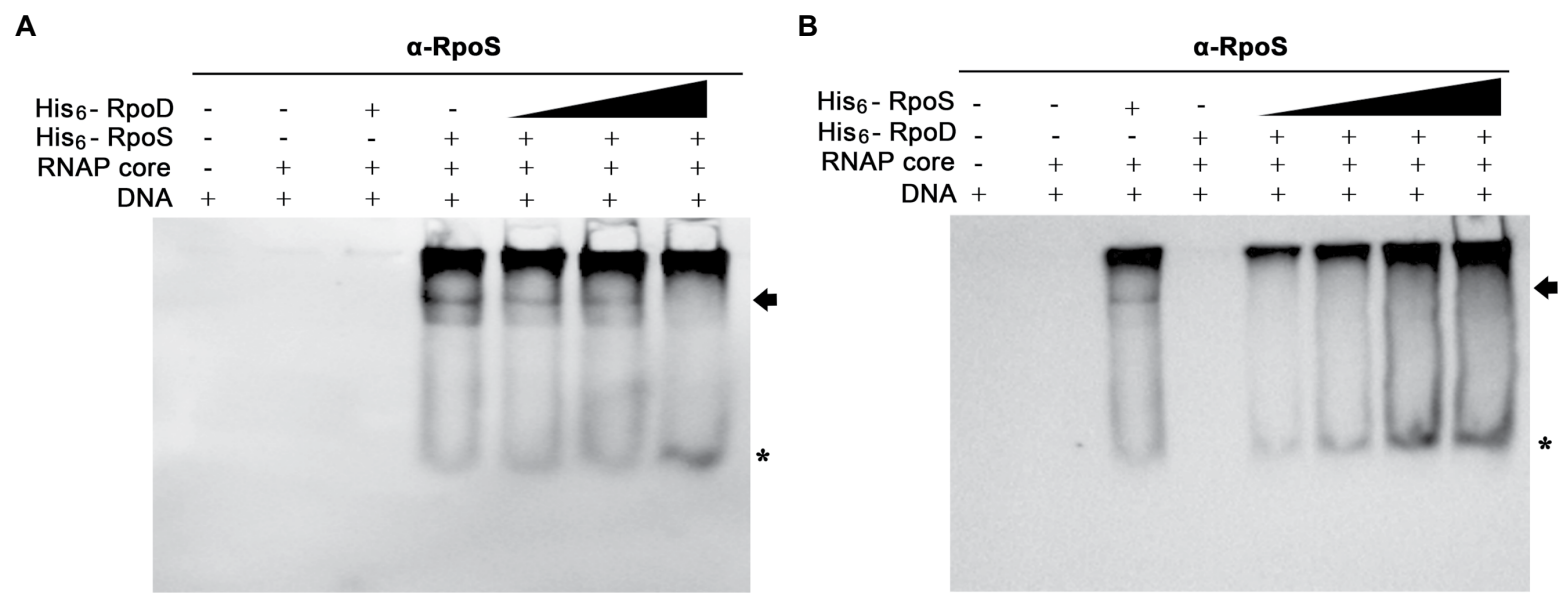
Competitive binding to the P*_ssrA_* region between σ^S^ and σ^70^ *in vitro*. **(A)** Competitive EMSA was conducted with His_6_-RpoS at a constant concentration of 75nM and with increasing concentrations (0, 12.5, 25, and 50nM) of His_6_-RpoD as a competitor. RNAP core enzyme was pre-incubated with different combinations of two σ factors and incubated with DNA containing the P*_ssrA_* region. DNA bands were stained using EtBr and shown in Supplementary Figure 3A. His_6_-RpoS was localized using subsequent immunoblotting with an anti-RpoS antibody. His_6_-RpoS comprising Eσ^S^ cross-linked with the P*_ssrA_* region is indicated with an arrow and free His_6_-RpoS is localized with an asterisk. **(B)** Competition EMSA was applied using His_6_-RpoD at a constant concentration of 75nM and with increasing concentrations (0, 25, 50, 100, and 150nM) of His_6_-RpoS as a competitor. After reconstitution of RNAP holoenzyme with different concentrations of σ factors, the interaction between RNAP holoenzyme and P*_ssrA_* region was analyzed using native gel electrophoresis followed by DNA staining (Supplementary Figure 3B) and immunoblotting with an anti-RpoS antibody. The location of His_6_-RpoS comprising Eσ^S^-P*_ssrA_* complex is indicated with an arrow and free His_6_-RpoS not associated with DNA is indicated with an asterisk.

The P*_ssrA_* DNA fragment was incubated with the core enzyme E and different concentrations of σ factors (His_6_-RpoS and His_6_-RpoD), and the levels of Eσ^S^ associated with the P*_ssrA_* region were determined using an anti-RpoS antibody. When His_6_-RpoS was used at a constant concentration of 75nM, but His_6_-RpoD was increased from 0 to 50nM, the band representing the complex between Eσ^S^ and P*_ssrA_* gradually diminished and disappeared at 50nM His_6_-RpoD (Figure 7A). On the other hand, when His_6_-RpoD was maintained at 75nM but His_6_-RpoS was increased from 0 to 150nM, Eσ^S^ failed to bind to the P*_ssrA_* region even at a 2-fold higher concentration of His_6_-RpoS than His_6_-RpoD (Figure 7B). This result suggests that the P*_ssrA_* region preferentially recruits Eσ^70^ when Eσ^S^ and Eσ^70^ are present at equivalent concentrations *in vitro*.

### Crl Promotes σ^S^ Competitiveness for Binding to the *ssrA* Promoter Region

**Figure 8:**
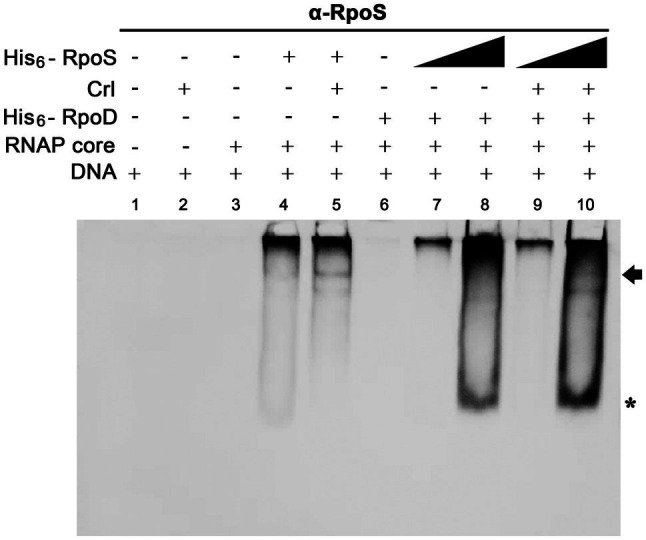
Effect of Crl on the binding affinity of σ^S^ to the P*_ssrA_* region *in vitro*. Competitive binding between His_6_-RpoS and His_6_-RpoD to the P*_ssrA_* region was re-examined in the presence of His_6_-Crl. In the absence of His_6_-RpoD (lanes 1–5), the RNAP core enzyme was incubated with 50nM His_6_-RpoS, which was pre-incubated with 280nM His_6_-Crl. In the competition between His_6_-RpoS and His_6_-RpoD (lanes 6–10), His_6_-RpoD was maintained at 50nM, while His_6_-RpoS was used at 25 and 280nM in combination with 280nM His_6_-Crl. RNAP holoenzyme was reconstituted using different combinations of His_6_-Crl, His_6_-RpoS, and His_6_-RpoD and incubated with P*_ssrA_* region DNA. After gel electrophoresis, DNA was analyzed by EtBr staining (Supplementary Figure 4), and His_6_-RpoS was localized using immunoblotting with an anti-RpoS antibody. His_6_-RpoS comprising Eσ^S^ cross-linked with the P*_ssrA_* region is indicated with an arrow, and free His_6_-RpoS is indicated by an asterisk.

For investigating the possibility that σ^S^ replaces σ^70^ and lowers the *ssrA* transcription, we searched for a co-regulator that could promote σ^S^ activity under stressful conditions and Crl was chosen as a candidate co-regulator of σ^S^-mediated *ssrA* transcriptional regulation. Crl is a small protein known to interact directly with σ^S^ *in vitro* (Bougdour et al., 2004). The P*_ssrA_* fragment was incubated with His_6_-tagged σ factors (σ^S^ and σ^70^) and Crl individually or in combination, and the σ^S^ bound to P*_ssrA_* was localized using an anti-RpoS antibody. In the absence of competition with σ^70^, Crl addition enabled σ^S^ (50nM) to form a complex between Eσ^S^ and the P*_ssrA_* region, whereas σ^S^ alone at 50nM were insufficient to form the Eσ^S^-P*_ssrA_* complex (Figure 8: compare lanes 4 and 5). In the absence of Crl, His_6_-RpoS even at 280nM was defeated in the competition with 50nM His_6_-RpoD and failed to form the protein-DNA complex (Figure 8, lane 8). However, pre-incubation of His_6_-RpoS with Crl rendered His_6_-RpoS competitive in forming the complex between Eσ and P*_ssrA_*, showing a shifted band (Figure 8, lane 10). Crl binding to σ^S^ might facilitate the formation of the RNAP holoenzyme incorporating σ^S^ instead of σ^70^, as suggested previously (Gaal et al., 2006; Typas et al., 2007a).

### σ^S^ Abundance Led to a Comprehensive Transcriptional Alteration of SPI-2 in Host Cells

**Figure 9:**
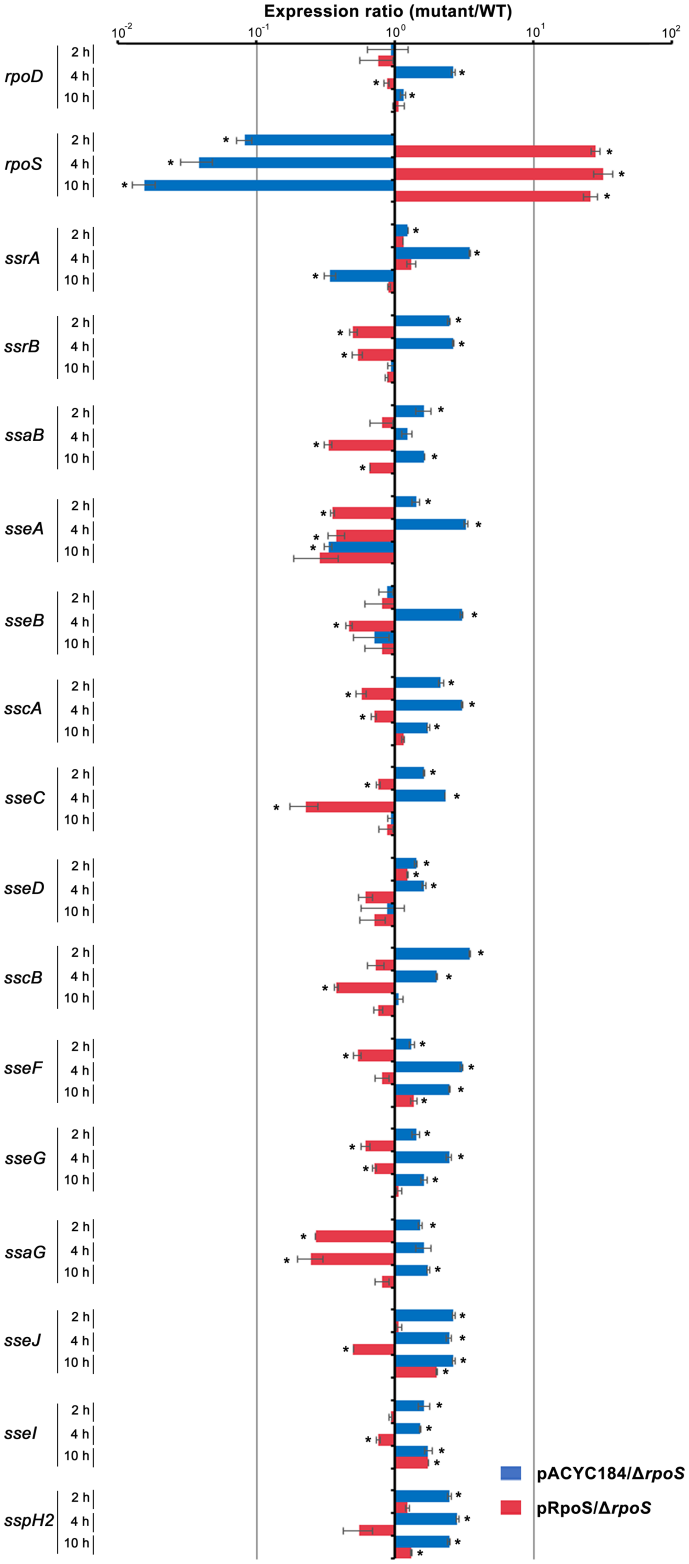
Transcriptional regulation of SPI-2 genes by σ^S^. *Salmonella* wild-type and Δ*rpoS* mutant strains containing pRpoS or pACYC184 were added to RAW264.7, and total RNA was isolated at 2, 4, and 10h post-infection and used to measure the transcription levels of genes, including *rpoD*, *rpoS*, and SPI-2 T3SS-associated genes. The Ct values for each gene were normalized to those of *gyrB*. Expression levels of each gene from Δ*rpoS* mutant strains containing pRpoS or pACYC184 were compared with those from wild-type strain containing pACYC184 and the fold-change (mutant/wild-type) was plotted. An asterisk indicates a value of *p*<0.05 in comparison with wild-type strain harboring pACYC184.

Our results comparing the ability of σ^S^ and σ^70^ to form the Eσ-P*_ssrA_* complex *in vitro* demonstrated that Eσ^70^ bound to the P*_ssrA_* region preferentially than Eσ^S^. Given the limited cellular resources of the RNAP core enzyme E, *ssrA* transcription may be dampened when σ^S^ stimulated by drastic stressors diverts the RNAP core enzyme E to its cognate regulatory circuit, which is critical for surviving the challenging stressors. SPI-2 genes controlled by SsrAB regulators are known to be activated under hostile conditions such as a nutrition-deprived environment and intracellular milieu (Beuzon et al., 1999; Deiwick et al., 1999), which are prone to stimulate σ^S^-mediated adaptation responses. We investigated the transcriptional response of SPI-2 genes when σ^S^ levels surged in response to stress and Eσ^S^-mediated transcriptional initiation overwhelmed the transcriptional activity of other Eσ complexes. *Salmonella* wild-type and Δ*rpoS* strains were added to macrophage cells, and the transcription levels of *rpoS*, *rpoD*, and SPI-2 genes were compared at 2, 4, and 10h after phagocytosis. σ^S^ was overexpressed by introducing pRpoS into the Δ*rpoS* mutant. The absence of *rpoS* increased *rpoD* transcriptional levels at 4h post-infection (Figure 9). Comparing mRNA levels of SPI-2 genes between wild-type and Δ*rpoS* strains showed that most SPI-2 genes increased their transcription in the absence of σ^S^ and addition of pRpoS nullified these alterations (Figure 9), indicating a negative role of σ^S^ in SPI-2 transcription. In accordance with the transcriptional regulation by σ^S^, the levels of T3SS2-associated proteins were decreased by the overexpression of σ^S^ (Supplementary Figure 6). However, the transcriptional response to σ^S^ abundance was different among the SPI-2 genes. Many genes, including *sscB*, *sseFG*, *ssaG*, *sseJ*, and *sspH2*, showed negative transcriptional regulation by σ^S^ abundance throughout the assay, whereas *sseCD* genes encoding the translocon components of T3SS2 (Chakravortty et al., 2005) showed minimal transcriptional alteration by σ^S^ abundance (Figure 9). Differential requirements among T3SS effectors depending on time and site during infection have been proposed in previous studies (Brawn et al., 2007; Nunez-Hernandez et al., 2014). The differential influence of σ^S^ between T3SS2-associated genes suggests that SsrA-mediated regulation is not the only mechanism by which σ^S^ participates in controlling SPI-2 T3SS-associated genes.

## Discussion

During host infection, *Salmonella* undergoes various stress conditions, such as gastric acidity, bile salts, oxidative stress, and nutrient starvation. Alternative σ factors are prominent regulatory proteins that enable bacteria to cope with diverse stresses by redirecting RNAP core enzymes to the transcription of genes required for survival and adaptation in these conditions. RpoS or σ^S^, a σ factor comprising the RNAP holoenzyme, is known to activate the transcription of genes associated with general stress resistance (Hengge-Aronis, 2002a). However, the regulatory roles of σ^S^ are not only restricted to stress-resistance genes. In *Salmonella*, σ^S^ was found to directly or indirectly control the expression of genes that make up more than 20% of the genome (Levi-Meyrueis et al., 2014; Lago et al., 2017), implying its multifaceted roles ranging from physiological remodeling against cellular damage to metabolic regulation of sugars, amino acids, and fatty acids (Ibanez-Ruiz et al., 2000; Lago et al., 2017). In addition, σ^S^ is involved in *Salmonella* virulence regulation. Rice et al. (2015) observed genes comprising SPI-1 and SPI-2, which are essential for *Salmonella* invasion into host cells and intracellular survival, were upregulated in the absence of σ^S^, indicating a negative role of σ^S^ in SPI-1 and SPI-2 expression. We found that σ^S^ could bind to the P*_ssrA_* region directly (Figure 5), and a surplus of σ^S^ repressed its transcription. This led to an overall downregulation of SPI-2 T3SS-associated genes (Figure 9). Direct negative regulation by σ^S^ was recently reported in the transcription of *esrB* in *Edwardsiella piscicida* (Yin et al., 2018). *Edwardsiella piscicida*, which is phylogenetically close to *Salmonella enterica*, exploits T3SS and T6SS to translocate virulence factors into host cells, and the expression of these virulence machineries is activated by the two-component regulatory system EsrAB, homologs for SsrAB in *S. enterica* (Wang et al., 2009; Yin et al., 2018). *Edwardsiella piscicida* σ^S^ was proposed to mediate a trade-off between stress adaptation and virulence by inhibiting *esrB* expression through a direct interaction between σ^S^ and the P*_esrB_* region (Yin et al., 2018). Binding of the Eσ complex to gene promoter regions is typically presumed to activate transcription initiation. Therefore, the negative regulation by σ^S^ may be attributed to the competition between σ factors for binding to the RNAP core enzyme (Farewell et al., 1998; Hsu, 2002). A surge in σ^S^ caused by bacterial adaptation to general stresses can exclusively occupy the pool of core enzyme E and impede transcriptional events mediated by other σ factors. However, alternatively to this passive regulation *via* competitive binding between σ factors, adhesion of the Eσ^S^ complex to promoter regions may sterically hinder binding of the Eσ^70^ complex and directly attenuate transcription, as demonstrated in *E. piscicida esrB* gene (Yin et al., 2018) and *S. enterica* serovar Typhimurium *sdh* gene (Levi-Meyrueis et al., 2015).

The promoter region of *ssrA* is occupied by multiple regulators, including HilD, SlyA, OmpR, and H-NS, and its transcription is controlled by the competitive binding of these regulators to the overlapping DNA (Banda et al., 2019). The consensus promoter sequences recognized by σ^70^ are also accessible to H-NS, whose binding blocks the access of Eσ^70^ and transcriptional activators such as OmpR. Anti-repressors such as HilD and SlyA relieve H-NS-mediated silencing by competitive binding to the P*_ssrA_* region (Banda et al., 2019). Considering the similar recognition motifs at −10 and −35 elements between σ^S^ and σ^70^ and the functional inter-compatibility between two σ factors in some genes, the downregulation of *ssrAB* by binding of σ^S^ to the P*_ssrA_* region can be achievable through several mechanisms. Firstly, binding of Eσ^S^ to the P*_ssrA_* region may not exert transcriptional initiation, but instead sterically hinder Eσ^70^-mediated transcription as demonstrated in the *E. piscicida esrB* gene (Yin et al., 2018). A σ factor associated with RNAP core enzyme directs transcription initiation at a specific promoter region but is assumed to dissociate upon transition from transcription initiation to transcription elongation because of a steric clash between the growing RNA product and the σ factor (Hsu, 2002). σ^S^ that is not released on time may impede promoter escape of the core enzyme E and hinder transcription elongation. Alternatively, the binding of Eσ^S^ to P*_ssrA_* region may produce incorrect transcripts, as shown in the transcriptional regulation of *crl* gene (Zafar et al., 2014). The *crl* gene with overlapping promoters sensed by two different σ factors of σ^70^ and σ^N^ may shut down its expression by association with Eσ^N^. σ^N^ increases in response to nitrogen limitation, forming a DNA-Eσ^N^ complex at the *crl* promoter region, but its binding results in a long noncoding RNA transcript lacking a ribosome binding site, thereby preventing Eσ^70^ from binding to the overlapped promoter and producing translatable *crl* mRNA (Zafar et al., 2014). We observed that DNA fragments bound to Eσ^S^ *in vivo* covered a long region from the known +1 site of *ssrA* transcript to the start codon for SsrA (Figure 5). To differentiate between these two possibilities, it is important to examine whether Eσ^S^ bound to the P*_ssrA_* region can lead to *ssrA* transcription and whether the resultant transcript can successfully be translated. Another possibility is the competitive Eσ^S^ binding among promoters with different binding affinities due to recognition motif preference and topological characteristics. Considering that the cellular σ^S^ concentration is low even in the stationary phase of growth (Jishage et al., 1996) and its affinity for RNAP core enzyme is the lowest among σ factors *in vitro* (Maeda et al., 2000), the P*_ssrA_* region occluded by multiple regulators may be less competent in recruiting Eσ^S^ and other promoter sites, which are preferentially responsive to σ^S^, may outcompete the *ssrA* promoter.

In order to cope with limited resources, bacteria allocate cellular resources between reproduction and maintenance in response to environmental cues. In the absence of nutrient depletion and hostile stressors, bacteria proliferate and deploy resources for reproduction. Under these favorable conditions, σ^70^ is exclusively used for the transcription initiation of housekeeping genes. On the other hand, bacteria challenged by stressful stimuli divert cellular resources to maintenance and resistance, replacing σ^70^ with alternative σ factors for comprehensive transcription alteration. σ^S^ orchestrates the expression of a large number of genes under conditions of starvation and general stress caused by pH, temperature, and osmolarity. SPI-2 T3SS and its cognate effectors critical for *Salmonella* intracellular survival and replication are thought to be induced by unfavorable stimuli encountered inside host cells (Lober et al., 2006; Fass and Groisman, 2009), which would also likely promote σ^S^-mediated stress adaptation processes. However, we observed that excessive σ^S^ production rather decreased the transcription of SPI-2 and its associated genes. Virulence effectors translocated *via* SPI-2 T3SS help intracellular *Salmonella* to compromise the host defense systems and facilitate intracellular proliferation and cell-to-cell spread (Grant et al., 2012; Jennings et al., 2017). However, overgrowth of *Salmonella*, which is less competent to manage hostile stresses, poses a disadvantage to long-term persistence inside hosts because the intense immune responses provoked by the proliferation may eliminate defective bacteria rapidly after all (Nunez-Hernandez et al., 2014). *Salmonella* executing σ^S^-mediated stress adaptation may attenuate aggressive virulence ascribed to SPI-2 to achieve a trade-off between stress adaptation and virulence. Notably, the growth rates of intracellular *Salmonella* vary depending on the infected cell types; *Salmonella* proliferated exclusively in CD18-expressing phagocytes *in vivo* (Richter-Dahlfors et al., 1997), while restraining its growth in non-professional phagocytes such as subepithelial fibroblasts (Cano et al., 2001). Therefore, the importance of SPI-2 T3SS for *Salmonella* survival varies depending on infection foci or cell types. For example, SifA, an effector translocated *via* SPI-2 T3SS, is essential for bacterial growth inside macrophages but is dispensable for survival inside fibroblast cells (Nunez-Hernandez et al., 2014). Interestingly, Grant et al. showed that *Salmonella* lacking SPI-2 T3SS remained inside phagocytes at a high replication rate but failed to leave the infected cells, suggesting a new role for SPI-2 T3SS in bacterial dissemination to other sites (Grant et al., 2012). Premature escape from infected host cells may impose unaffordable expenses on *Salmonella* to resist severe host defense systems and constrain its successful host colonization. In this context, it is an energy-effective strategy for *Salmonella* to employ σ^S^ as a dual-purpose regulator that aids in adaptation and resistance against unfavorable conditions and lowers unnecessary virulence attributable to SPI-2 at the same time.

## Data Availability Statement

The original contributions presented in the study are included in the article/Supplementary Material, further inquiries can be directed to the corresponding author.

## Author Contributions

SK designed and conducted the experiment and interpreted the data. EK performed and analyzed the experiment. HY conceived and coordinated the study. SK and HY wrote the manuscript. All authors contributed to the article and approved the submitted version.

## Funding

This study was supported by a grant (2019R1A6A1A11051471) from the Priority Research Centers Program funded by the National Research Foundation of Korea (NRF) and a grant (2021R1F1A1058498) of the Basic Science Research Program through the NRF funded by the Korean government (MSIT).

## Conflict of Interest

The authors declare that the research was conducted in the absence of any commercial or financial relationships that could be construed as a potential conflict of interest.

## Publisher’s Note

All claims expressed in this article are solely those of the authors and do not necessarily represent those of their affiliated organizations, or those of the publisher, the editors and the reviewers. Any produ that may be evaluated in this article, or claim that may be made by its manufacturer, is not guaranteed or endorsed by the publisher.

## Footnotes

^1^ https://imagej.nih.gov/ij/
