# Supplementary material for: σS-Mediated Stress Response Induced by Outer Membrane Perturbation Dampens Virulence in Salmonella enterica serovar Typhimurium
Source: Front Microbiol. 2021 Sep 30;12:750940. doi: 10.3389/fmicb.2021.750940 (PMC8516096; doi:10.3389/fmicb.2021.750940)
Supplement: Supplementary file 1 [file Data_Sheet_1.docx]

**Supplementary Material**

# Supplementary Tables and Figures

## Supplementary Tables

# Table S1. Primers used in strain and plasmid construction

| **Primers** | **DNA sequence from 5' to 3'** |
| --- | --- |
| sseC-HARF | CTATAGTAGCGTTCAGGCGAGAATAGCTGGCTATCGCGCTATTCCGGGGATCCGTCGACC |
| sseC-HARR | GTGCTACGTTACTCGCTTCCATATTTTTATCCTCAGATTAGTGTAGGCTGGAGCTGCTCC |
| sseC-DF | AGATAGAACAAAAACGCTTAGAGGAG |
| sseC-DR | ATATCGCGCAGCTTTTTGGCAAG |
| ssaN-HARF | CGAGCTACTTATAGAAAAATTACACCAAATACTCACCGAGATTCCGGGGATCCGTCGACC |
| ssaN-HARR | TCACGCCGCGCGATTATCTCCAGCAAAGTTTCCATGATCAGTGTAGGCTGGAGCTGCTCC |
| ssaN-DF | AGGTTGAACTGTTAATACGCATTG |
| ssaN-DR | CCATTAATTCTTTCAGTCTGGTAG |
| pRpoS-CF | AAAGGATCCTTGCGTCTCAACCAACAATTAC |
| pRpoS-CR | AAAGTCGACTTACTCGCGGAACAGCG |
| pACYC184_seq-F | AGCCCCATACGATATAAGTTG |
| pACYC184_seq-R | TCCTACGAGTTGCATGATAAAG |
| pRpoS-CF2 | AAAGAATTCCGTCAAGGGATCAC |
| pRpoS-CR2 | TTTAGATCTTTACTCGCGGAACAG |
| pBbA2sk-RFP-Seq-F | ATGCCAATACAATGTAGGCTG |
| pBbA2sk-RFP-Seq-R | TTTGGTAACTTTCAGTTTAGCG |
| pssrA-lacZ-CF | TTTGAATTCATCCCTCCTCAGACATAAATGGG |
| pssrA-lacZ-CR | AAAGGATCCTATACTAAAGATGTTTGCAGCGTATTC |
| pssrB-lacZ-CF | TTTGAATTCTTGAAAATGCCGTATCGGCTGGAG |
| pssrB-lacZ-CR | TTTTATGTCAGGCTCGTATGCACAAC |
| pRS415-Seq-R | TGCTGCAAGGCGATTAAGTTG |
| RpoS-6His-F | GACGTGGATCCCATCACCATCACCATCACAGTCAGAATACGCTGAAAGTTC |
| RpoS-6His-R | TTTAAGCTTTTACTCGCGGAAC |
| RpoD-6His-F | TGAGAGGATCCCATCACCATCACCATCACCCGCATATCGATCGGGAAGC |
| RpoD-6His-R | TTTAAGCTTTTAATCGTCGAGGAAGCTG |
| Crl-6His-F | TGAGAGGATCCCATCACCATCACCATCACACGTTACCGAGTGGACAC |
| Crl-6His-R | TTTAAGCTTTTATGCCGACAGTTTTACC |
| pUHE21-lacI^q^ Seq-F | AGATTCAATTGTGAGCGGATAAC |
| pHUE21-lacI^q^ Seq-R | GGTCATTACTGGATCTATCAAC |

**Table S2. Primers used in qRT-PCR**

| **Primers** | **DNA sequence from 5' to 3'** |
| --- | --- |
| gyrB-qRT-F | TCGCTCAGCAGTTCGTTCAT |
| gyrB-qRT-R | GATTGCGGTGGTTTCCGTAA |
| rpoD-qRT-F | AATGCTCCGTTGCCGAATAC |
| rpoD-qRT-R | ATCGACAAAGCCGGTGATAAGA |
| rpoS-qRT-F | TGAAGAGGAACCCAGTGATAACG |
| rpoS-qRT-R | CCCAATCTCACCAAGGTAAAGC |
| ssrA-qRT-F | GCGTACTCGGTGCAATTGAA |
| ssrA-qRT-R | AGACTCGATGCGTGAAAAATCC |
| ssrB-qRT-F | GCTTTTCGCTGATCCCATGA |
| ssrB-qRT-R | CAACGTTGAATCGGGAAGCT |
| ssaB-qRT-F | GGGATTCATGCTGGCAGTTT |
| ssaB-qRT-R | CAGGATGCCCATCAATAGTCATT |
| sseA-qRT-F | AAGGCTGCGTTTAGTGAATATCG |
| sseA-qRT-R | CCCTTTCAGCAAGCTGTTGACT |
| sseB-qRT-F | GGAGGTGCCTGAGGATGTAATT |
| sseB-qRT-R | AGCTTCCCATGATCGCCATA |
| sscA-qRT-F | AGCGCGACTTTTTCAATTGC |
| sscA-qRT-R | TTCCCCCCAATGTTTTTGAG |
| sseC-qRT-F | TTAAAGCCGGAGCCGAAA |
| sseC-qRT-R | CGACGGCTTCACAACCAAAT |
| sseD-qRT-F | ATGCGCAGCTATAACGTAGAAAAA |
| sseD-qRT-R | TGCTCTAAACGCTTCATCAATTG |
| sscB-qRT-F | AGCCATCCAGAACCGGTTTA |
| sscB-qRT-R | GCATCCGCATAACTCATCTTGA |
| sseF-qRT-F | CAGCAAAAGGAGCCATTACAAA |
| sseF-qRT-F | AGCAAGGGTGTTAGCGCAGTTA |
| sseG-qRT-F | GGACTTGCGAAACGAGTGATAGA |
| sseG-qRT-R | CCAGCGGAACAGCATAAAATC |
| ssaG-qRT-F | GGCCAGGCCATTAATGACAA |
| ssaG-qRT-R | GGCAAATTGCGCTTTAATCATC |
| sseJ-qRT-F | TCACATCCCAAGCCCAAAAA |
| sseJ-qRT-R | TTCCGCCAAAGTATTGACCAT |
| sseI-qRT-F | TGGATGAAGGCAGTCAGGAGAT |
| sseI-qRT-R | GTTTCACTGTACCCCTGGCAAT |
| sspH2-qRT-F | TGATACCCCCCCTGAAATGA |
| sspH2-qRT-R | GTGACAAATCGTCCAGATGCA |
| ssaD-qRT-F | GGTGGAATGGGTGTCCTGTTAA |
| ssaD-qRT-R | CGCCCGCACATAATGAATATT |
| ssaK-qRT-F | GAGGCATTGATGCGAGAAACT |
| ssaK-qRT-R | CGGCATATCGTGTTGAGGAA |
| hilA-qRT-F | GCTGCACCAGGAAAGCATTAAG |
| hilA-qRT-R | CGAAGTCCGGGAATACATCTGA |
| hilC-qRT-F | GCCGCTGAAGAGGTGAGTTTTA |
| hilC-qRT-R | AATATTTCCAGCCCCCATACG |
| hilD-qRT-F | GCTGTTCCTGCTTACTGCTTTTC |
| hilD-qRT-R | AATGTTGTAAACGCGCTCCTTT |
| invF-qRT-F | GCGGAAAAGCGAAGAGTGAA |
| invF-qRT-R | AACGGCTAATTGGGTGATGTTC |
| rtsA-qRT-F | CGCAAAACTGGCAGAGGAAA |
| rtsA-qRT-R | CCGGACGATGTCGTAATTGA |
| invA-qRT-F | ACAAAACATATGCTGGACCAACTG |
| invA-qRT-R | ACGCTGCAAAACTTCAGATATACG |
| sopA-qRT-F | TACGTCACAAAGCCAACCTCTCT |
| sopA-qRT-R | GTGGCATTTGCAGCCAGATA |
| sirA-qRT-F | AAAAGAATTTCGTCTCGCCC |
| sirA-qRT-R | CATTGTTACTGGCATTCGGA |
| barA-qRT-F | GGAGTCTATCGGCCAACTTATCA |
| barA-qRT-R | GGTCCAGATGGAAATTAGACGTTAC |
| phoB-qRT-F | CCCGTAGAAGCCGAAGATTATG |
| phoB-qRT-R | CATGGCTTCACGTTTGAGATGT |
| phoR-qRT-F | TACTGCGTTATCCGGAGTTTACC |
| phoR-qRT-R | ATAAGGCATGACGCGGATCT |
| phoP-qRT-F | AGATGCCAGGGAAGCTGATTAC |
| phoP-qRT-R | CCAGCGGCGTATTAAGGAAA |
| phoQ-qRT-F | CCTCGCCAAATGGGAAAATA |
| phoQ-qRT-R | TTTGCCCGTTTCATCGTAAAT |
| hilE-qRT-F | AACCACAACCCGACAAAACG |
| hilE-qRT-R | TAGTGCTCATAGAGGCCTGGTGTA |
| lon-qRT-F | CAGTCCATCGCTAAAGCAACAG |
| lon-qRT-R | TGGATCAGTTTACCCGGCATA |
| hha-qRT-F | GGAGCGCGTCATTGAGAAAA |
| hha-qRT-R | GGGATCTTGTCGTAGAGTTTGTTCA |
| fur-qRT-F | TCACGTCAGTGCGGAAGATTT |
| fur-qRT-R | GGCATCGTCAAACTGGTTCA |
| hns-qRT-F | GACCCGAATGAACTGCTGAATAG |
| hns-qRT-R | TGGCCAGTCCAGGTTTTAGTTT |
| csrA-qRT-F | ATGCTGATTCTGACTCGTCGA |
| csrA-qRT-R | GATGGACAGAAACTTCTTTCGG |
| csrB-qRT-F | AGTCGTACAACGAAGCGAAC |
| csrB-qRT-R | TCCTGGAGGTGTCCTTTAAC |
| rcsA-qRT-F | AAGGGAAATCAACGACATCGA |
| rcsA-qRT-R | TGTTGACTGTCTGAAGGCGTATG |
| rcsB-qRT-F | GATAAGCGTCTGTCGCCAAAA |
| rcsB-qRT-R | TTTCTTCTGGCTGCTGATGGT |
| envZ-qRT-F | ATTTTTCTCCGCTTTTCCGTTA |
| envZ-qRT-R | CGGCATGTTCAAGATCCACTAA |
| ompR-qRT-F | TGACCCGTGAATCTTTCCATCT |
| ompR-qRT-R | TGATCGGCATTGGATTACTTTG |
| fis-qRT-F | CGGTTAAACAGGCACTGAAGAA |
| fis-qRT-R | ACGGGTGTATTGCATCACCAT |
| fliZ-qRT-F | CTGCTGATGCGCTTTATATTGC |
| fliZ-qRT-R | TGCAGGACGGTTTTCTCGAT |
| rpoE-qRT-F | CGATTGAAGCAGAAAACTTTGAAA |
| rpoE-qRT-R | ATCTTCCGGGAGGGACTCAATA |

**Table S3. Primers used in ChIP-(q)PCR and EMSA**

| **Primers** | **DNA sequence from 5' to 3'** |
| --- | --- |
| PssrA-ChIP-F | ACAGGCGATTCTATCATTCG |
| PssrA-ChIP-R | ATAATGCTTCCCTCCAGTTG |
| PssrB-ChIP-F | AATCACTGGACCTCTTGCTG |
| PssrB-ChIP-R | TTTCATTTTGCTGCCCTCGC |
| R1-F | AAATGGGAGTTTCTATCAAATTCG |
| R1-R | AGACAGCATCCTGATATTGTAC |
| R2-F | GTCTACATATACCTTGTCACAG |
| R2-R | TTGTAAGTTTTTATGTCAATGCTG |
| R3-F | TTGACATAAAAACTTACAATTTG |
| R3-R | ATATAACCCAGTCGATGACTAC |
| R4-F | GTCATCGACTGGGTTATATATG |
| R4-R | CTTTGGCACTTGATCACTATC |
| R5-F | TCAAGTGCCAAAGATTTTGCAAC |
| R5-R | CAGATAGCAGCCAAATAATTATTG |
| ssrA-EMSA-F | GACATAAAAACTTACAATTTG |
| ssrA-EMSA-R | AGTACATATAGTTTCATCAG |
| csgBA-EMSA-F | ATACTTTGGTATGAACTAAAAAAGAA |
| csgBA-EMSA-R | CTGGTCGTACATTTAAGAAATT |
| STM14_1978-EMSA-F | TGCCACAGATGTTGCGATTT |
| STM14_1978-EMSA-R | TGACATTGCCTGCTTCGTTATT |

## Supplementary Figures





**Supplementary Figure 1.** Expression of SPI-2 genes in Δ*ycfR* mutant after macrophage infection and in AMM broth condition

(A) Expression of SPI-2 genes was analyzed in intracellular *Salmonella* strains (wild-type and Δ*ycfR* mutant) at 10 h post-infection of RAW264.7 cells. (B) Transcription levels of SPI-2 genes were compared between wild-type and Δ*ycfR* mutant strains cultivated in AMM broth for 3 h, mimicking the intracellular conditions. The Ct values of qRT-PCR were normalized using those of *gyrB* and a fold-change (Δ*ycfR* /wild-type) was shown with an asterisk indicating P-value < 0.05.

**

**

**Supplementary Figure 2.** Expression of 21 regulators in *Salmonella* lacking YcfR

Transcription levels of 21 regulator genes were compared between wild-type and Δ*ycfR* mutant strains cultivated in LB medium broth for 10 h. qRT-PCR Ct values of each gene were normalized using those of *gyrB* and an expression fold-change between wild-type and Δ*ycfR* mutant strains was plotted. An asterisk indicates a difference of P-value < 0.05.

**

**

**Supplementary Figure 3.** Promoter region of *ssrB* and the primer scheme used in ChIP-PCR

The +1 site of *ssrB* transcription is indicated by a broken arrow, and its start codon is in bold. The region amplified by ChIP-PCR is indicated by a black dashed line.





**Supplementary Figure 4.** Competitive EMSA experiments using different concentrations of His_6_-RpoS and His_6_-RpoD

(A) Competitive EMSA was conducted with His_6_-RpoS at a constant concentration of 75 nM and increasing concentrations (0, 12.5, 25, and 50 nM) of His_6_-RpoD as a competitor. RNAP core enzyme was pre-incubated with different combinations of two sigma factors and incubated with DNA containing the P*_ssrA_* region. After electrophoresis using 5% native polyacrylamide gel, DNA bands were stained with EtBr and visualized using the ChemiDoc MP System. (B) Competition EMSA was applied using His_6_-RpoD at a constant concentration of 75 nM and increasing concentrations (0, 25, 50, 100, and 150 nM) of His_6_-RpoS as a competitor. After reconstitution of the RNAP holoenzyme with different concentrations of sigma factors, DNA containing the P*_ssrA_* region was added, and the interaction between the RNAP holoenzyme and P*_ssrA_* region was analyzed using native gel electrophoresis followed by DNA staining.

**

**

**Supplementary Figure 5.** Competitive EMSA experiments using different concentrations of His_6_-RpoS and His_6_-RpoD in the presence of Crl

Competitive binding between His_6_-RpoS and His_6_-RpoD to the P*_ssrA_* region was re-examined in the presence of His_6_-Crl. In the absence of His_6_-RpoD (lanes 1 to 5), the RNAP core enzyme was incubated with 50 nM His_6_-RpoS, which was pre-incubated with 280 nM His_6_-Crl. For assessing the competition between His_6_-RpoS and His_6_-RpoD (lanes 6 to 10), His_6_-RpoD was maintained at 50 nM, while His_6_-RpoS was used at 25 nM and 280 nM in combination with 280 nM His_6_-Crl. RNAP holoenzyme was reconstituted using different combinations of His_6_-Crl, His_6_-RpoS, and His_6_-RpoD and incubated with P*_ssrA_* region DNA. After electrophoresis using polyacrylamide gel, DNA bands were stained with EtBr and visualized using the ChemiDoc MP System.

**

**

**Supplementary Figure 6.** Expression of SPI-2 proteins in *Salmonella* overproducing σ^S^

*Salmonella* wild-type strains harboring pRpoS2 or pBbA2sk-RFP were cultivated in LB medium broth containing aTc at 0.5 ng/ml for 8 h and the expression levels of SPI-2 proteins (SseC and SsaN) tagged with HA and DnaK were measured using immunoblot assay. HA-tagged proteins are marked with arrow heads. The cytosolic protein DnaK was used as a control to normalize the protein amounts between the lanes. ▶
